# Venomous Snakes—A Request

**Published:** 1897-12

**Authors:** Alex W. Sterling

**Affiliations:** M. D., M. B., C. M., (Edin.) D. P. H., (Lond.); Atlanta, Ga.


### VENOMOUS SNAKES—A REQUEST.

BY ALEX W. STERLING, M. D., M. B., C. M., (Edin.) D. P. H., (Lond.)
ATLANTA, GA.

It is probably well known that Dr. Calmette of Lyons and Dr. Fraser, professor of Materia Medica in the University of Edinburgh, have for some time been endeavoring to produce a cure for poisoning by snake-bite by the production of an *attenuated virus* for hypodermic use, and that in the laboratory at least their efforts have been crowned by considerable success. It may not, however, be so well known that Dr. Fraser has quite recently (1) been experimenting with *bile* with the same object in view. He first employed the bile from the snake itself, and found that a minute quantity injected under the skin of animals prevented the lethal effects of the venom. The active part of the bile he was able to separate by dissolving in water; it is insoluble in alcohol. He then proceeded to experiment with the bile of other animals, and discovered that that also possessed the desired power even in minute quantities, though larger than those found sufficient when snake bile was employed. He ventured to deduce from these results that the same constituents of the hepatic secretion would be found capable of destroying the toxins of diseases such as diphtheria, tetanus, etc. Dr. Fraser has since given by subcutaneous injection (2) to a rabbit 0.15, C. CM. per kilo of diphtheria toxin mixed with 0.05 gramme per kilo of the dried bile of normal rabbit, and to a second rabbit 0.15 C. CM. per kilo of

1 British Medical Journal, July 17, 1897.
2 British Medical Journal, September 4, 1897.

diphtheria toxin, mixed with 0.025 gramme per kilo, of rabbit's bile; no bad symptoms ensued, except a slight rise in temperature, and a month later both animals had gained in weight. Three other rabbits received severally 0.15, 0.75 and 0.05 C. CM. per kilo of the same toxin, and they were all dead within ten days.

When we consider that some 17,000 persons die annually in India alone from snake-bite, and that throughout the world the number is probably many times that figure, and when we further consider what a deterrent the fear of snake-bite is, we get some idea of the importance of such work as that which is being carried on by Professor Fraser. (I say nothing further here of it for more important relationship to diphtheria, tetanus, etc., as that lies without the scope of this communication.)

My principal object in writing now is to bring before the profession a request which I have received from Professor Fraser that I would endeavor to procure for him the poison gland and gall bladders of as many snakes as possible, for his ability to continue his investigations is limited by the difficulty of obtaining the necessary material. I shall here set down his directions for the removal of those parts of the snake's body in order that any one who may have an opportunity of procuring recently killed snakes may, if he will be good enough to take the trouble, prepare the parts for transportation, and to aid in what may prove a very useful work.

The venom glands are situated immediately beneath the skin of the upper jaw, below and posterior to the orbits, and can easily be exposed, their ducts tied, and the glands then dissected out. They can then be hung up in warm (not hot), and if possible dry atmosphere, in which they soon become perfectly dry.

The gall bladder is particularly large in venomous snakes, but when dried with its contents shrinks into a small bulk. It is situated in front, and a good way nearer the tail than the middle of the snake. The duct is long and can easily be tied. The dried venom gland and gall bladder of each snake should be tied together and labelled, giving the name of the snake. If there is any doubt regarding the name it would be well to dry the head after removal of the venom glands, and send it also.

Dr. Fraser has expressed his willingness to pay for these, and if the parts are sent to me I shall make all arrangements necessary. I trust that what has been said will be sufficiently interesting to induce some members of the profession who live in districts in which snakes are to be found to use their influence with such members of their community as may have a predilection for snake-hunting in order that Professor Fraser's request may meet with an adequate response.

